# The Value of Playwork for Care Home Residents Living With Dementia: A Pilot Study

**DOI:** 10.1177/14713012251362271

**Published:** 2025-08-02

**Authors:** Chloé Bradwell, Mike Wragg, Nicky Everett

**Affiliations:** 1 School of Health, Leeds Beckett University, Leeds, UK

**Keywords:** care homes, dementia, play, playwork, intergenerational, playfulness, relation-centred care, dementia citizenship

## Abstract

Playwork is a profession that focuses on enabling and enriching children’s play experiences, creating a space for spontaneous, self-directed play. The application of playwork principles to dementia care holds promise and resonates with a relational approach to care. However, this area of practice has not yet been explored. This study aimed to explore if and how playwork approaches could be applied with people living with dementia and their impact on residents and those delivering the programme. A five-week playwork programme, delivered by undergraduate playwork students and lecturers, was piloted in a care home, with residents living with dementia. Interviews were conducted with care home staff, students, and playwork lecturers, and reflective diaries of the playwork sessions were maintained by students and lecturers. The findings indicate that playworkers can feasibly adapt their approaches so they are appropriate for older adults living with dementia. Playworkers can encourage agency and support free expression and exploration for residents. The sessions were perceived as having a positive impact on residents’ emotional wellbeing, sense of recognition, social interaction, and engagement, as well as on some staff members’ assessments of residents’ abilities. The study also highlights the crucial role of care staff expertise during the sessions, particularly in addressing the medical and physiological needs of residents. However, engaging care staff proved challenging, resulting in a lack of continuity after the project concluded.

## Introduction

In the UK, over 400,000 people live in care homes, with 70% experiencing dementia or memory problems (Berg, 2024). For those individuals, moving into an institutional setting often leads to significant emotional and psychological challenges, such as a loss of independence, reduced social interaction, and increased feelings of isolation (Lapane et al., 2022). Current care models often fall short in addressing the holistic wellbeing of residents, particularly those living with dementia. In response, scholars are increasingly calling for a more comprehensive approach that prioritises authenticity, playfulness, and spirituality in supporting individuals (Britt et al., 2023; Latham et al., 2020). This paper will consider how playwork might offer an avenue to address some of the needs of care home residents living with dementia.

Playwork is a method for working with children that supports and facilitates their play. It is also the name of the profession that facilitates this method. Playwork is unique among child-focused professions in that it takes an asset-based approach, aiming not to produce predetermined developmental outcomes or change a child’s behaviour, but to support their self-directed play. Playworkers regard playing as a bio-psychological drive, which if suppressed may result in physical and emotional harm to the child (Brown et al., 2018). Playworkers aim to support children in taking full control over the content and purpose of their play. While they recognise that play can bring many immediate and long-term developmental benefits, they believe these benefits are greatest when adults interfere the least. In other words, the more children lead the play themselves, the more they gain from it (Brown, 2018).

Playwork originated in the post-war adventure playground movement and shares core values with the children’s liberation movement of the 1960s. At its heart is a non-judgemental acceptance of children as they are. This is closely tied to the idea of ‘unconditional positive regard’ (Rogers, 1951), which means relating to children with consistent respect, trust, and support, regardless of their behaviour. In doing so, playworkers embrace a view of the child that challenges dominant, adult-centred narratives and practices. This approach involves a conscious effort to give up adult control, set aside preconceived ideas, and follow the child’s lead. Playworkers create flexible environments where children are free to play in ways they choose —recognising that play which may seem risky or challenging to adults can offer especially valuable benefits (Brown, 2018).

Although often associated with childhood, play is a fundamental aspect of human life, providing joy, connection, and cognitive stimulation across all ages (Proyer, 2012). The principles underpinning playwork, such as unconditional positive regard, resistance to prescriptive outcomes, and the creation of permissive, flexible environments (Brown, 2014) resonate strongly with the needs of people living with dementia, particularly those in care home settings. By adapting a playwork-informed approach, care practices could be reoriented to support residents’ agency, emotional wellbeing, and social engagement, not through structured activities alone, but by creating conditions in which spontaneous, self-directed moments of playfulness are recognised and valued.

The application of play and playfulness is already present in dementia care through arts-based programmes such as therapeutic clowning, humour therapy, and performance-based activities (Haire & MacDonald, 2021; Houben et al., 2020; Kontos et al., 2016; Swinnen & de Medeiros, 2018; Treadaway et al., 2019). These methods allow individuals with dementia to express themselves in ways that transcend the limitations of cognitive decline, building on their ability to create and connect in the present moment. Similarly, playwork’s emphasis on agency and creative expression could provide a platform for residents with dementia to engage in meaningful activities that promote emotional wellbeing and social engagement. By fostering an environment where residents are free to dictate the course of their play, playworkers could address some of the key emotional and psychological needs of people with dementia. For example, while an activity coordinator might organise a card-making session in the weeks prior to Christmas, a playworker would only provide a range of materials with the potential to make a Christmas card and create a social environment in which individuals felt empowered to engage.

Despite possible benefits, playful programmes (e.g. doll therapy and therapeutic clowning) have also been subject to criticism for potentially being demeaning or infantilising (Chinnaswamy et al., 2021; Jongsma & Schweda, 2018; Kenning, 2018). Critics argue that these approaches may undermine agency and dignity by reinforcing stereotypes that reduce individuals with dementia to childlike roles rather than recognising them as adults with complex emotional and social needs. Playwork, with its focus on self-directed, open-ended play, may provide a framework that is both respectful and empowering to people living with dementia. However, to date, playwork as a professional approach has not been applied in dementia care settings, and its potential impact on residents remains unexplored. Given the mixed perceptions of play in dementia care and the ongoing debate over the appropriateness of various playful approaches, a study examining whether playwork can be adapted for use with people living with dementia is both timely and necessary.

This paper evaluates the implementation of a five-week playwork-based programme in a care home, conducted in collaboration with final-year Childhood Development and Playwork BA (Hons) students, care home staff, and residents. The study draws on data from focus groups and interviews with care home staff, students, and playwork lecturers, alongside an analysis of reflective diaries maintained by students and lecturers. It explores the key playwork approaches employed, how these were adapted for residents living with dementia, and the perceived impact of these approaches on the residents.

## Playwork Programme

### The Care Home

The project took place in a privately owned residential care home in the North of England, which provides care for up to 40 residents. At the time of the research, most residents were living with dementia. This home was selected due to its focus on dementia care and its expressed desire to provide innovative approaches to engagement. At the time of the study, the Care Quality Commission (CQC) had rated the home as requiring improvement. Additionally, the COVID-19 pandemic had significantly disrupted the home’s activities and its connections with the local community. In response, the management was keen to rebuild these relationships and saw participation in the pilot as offering new positive opportunities for residents and staff.

### Programme Participants

The programme was led by two senior Childhood Development and Playwork lecturers and four third-year student volunteers. Many entered the project with assumptions shaped by societal narratives that depict dementia care as predominantly restrictive, medicalised, and lacking in meaningful engagement. Their participation in this project provided a rare opportunity to work in a care setting and challenge their preconceptions through firsthand experience.

**Figure 1.**
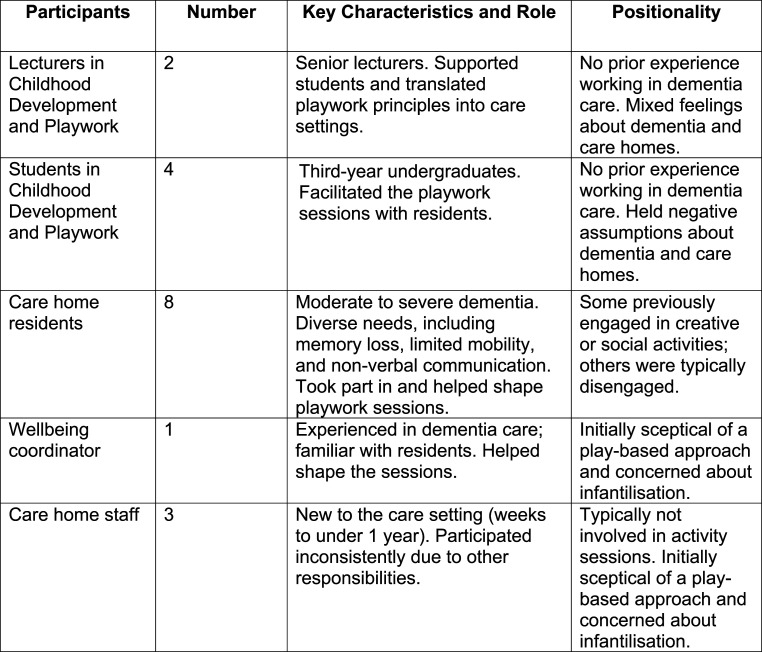
Participants Overview

The programme involved the regular participation of eight care home residents living with moderate to severe dementia (see Figure 1: Participants Overview). Participants had a range of needs and experiences, including memory loss, mobility limitations, visual impairment, and non-verbal communication. While some had a history of engaging in creative or social activities, others typically did not participate in the care home’s group sessions. Six residents were invited by the wellbeing coordinator, who believed they would respond well to the play-based approach. Two additional residents chose to join after observing the first session; an unexpected development, as they were usually non-verbal or reluctant to engage in group activities.

A member of staff responsible for resident activities and wellbeing was involved in piloting the programme. They brought valuable dementia care expertise and insight into residents’ preferences and needs. Initially, they expressed reservations that a play-based approach might be perceived as infantilising and that many residents would be unable or unwilling to participate in the activities.

Other care home staff members joined the sessions on an ad hoc basis, but their participation was inconsistent due to frequent interruptions as they were called away to attend to other responsibilities. Staff who took part were relatively new to the care setting, with experience in their current role ranging from a few weeks to under a year. They did not usually join in the activities provided in the home, other than to provide care for residents.

### The Playwork Sessions

**Figure 2.**
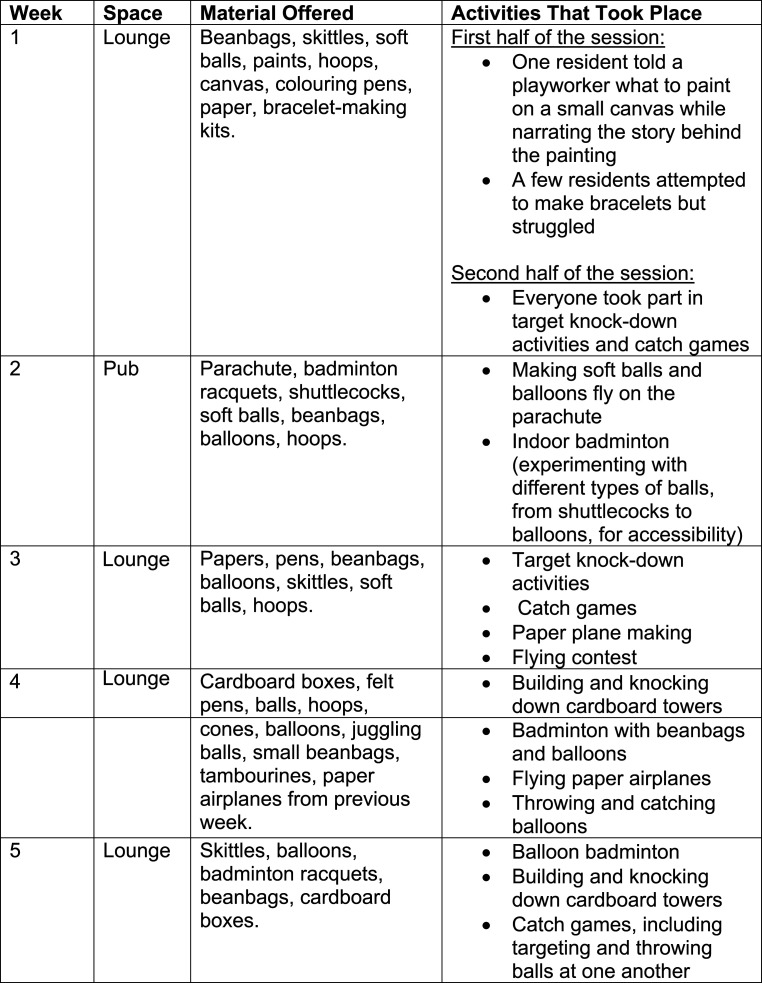
Sessions Overview

The playworkers conducted five in-person sessions, held weekly between April and June 2024 (see Figure 2: Sessions Overview). Four sessions took place in a private lounge, and one was in a small, pub-themed area. As this space could not accommodate everyone, the playworkers extended into a nearby corridor, which had a seating alcove for safe use.

Two months before the project began, the students and lecturers attended a Dementia Friends session (2024) — a one-hour workshop led by an Alzheimer’s Society ambassador to raise awareness about dementia and how to support individuals with the condition. They also met with the home manager and the wellbeing and activity coordinator to develop session plans and visit the care home. The playworkers proposed a variety of activities using open-ended materials, or ‘loose parts’ (Brown, 2018), to encourage diverse play behaviours, including gross motor skills. Based on prior experience, the care home staff, however, suggested focusing sessions on arts and crafts and so this recommendation was trialled for the initial session. Due to poor engagement during the first session, the playworkers returned to their original plan — introducing more active, play-based opportunities and activities focused on gross motor skills.

Sessions were planned weekly, adapting flexibly to the residents’ needs and preferences. Materials were laid out for residents to choose how they wished to interact. The table below outlines the materials provided and their use during the sessions.

## Evaluation Methods

### Data Collection

The evaluation methods chosen for this study were designed to capture the complexity and nuance of implementing playwork in a care home setting while respecting ethical considerations related to consent and capacity. No data were collected from care home residents as they did not have the capacity to consent (see *3.4 Ethics for further information*). A qualitative approach allowed for in-depth exploration of participants’ experiences, perceptions, and reflections (Braun & Clarke, 2013). It included:

#### Reflective Diaries

Students and facilitators were asked to keep a reflective diary throughout the evaluation. Diary methods allow for the collection of data on unique individual perspectives of social, psychological, and physical processes as they occur over time (McCombie et al., 2024; Olorunfemi, 2023). Participants were given the choice of written or audio format to ensure inclusivity. The focus was on the implementation of the sessions and perceived impact, capturing more detailed features such as what worked well or could have been more effective. The reflective diary data set consisted of 30 written entries from 4 students and 2 lecturers over the five-week period, with each entry ranging from 500 to 1,500 words.

#### Group Reflections

Three group reflections with the playwork lecturers and students were conducted in person following sessions two, three, and four. Each reflection lasted between 30-45 minutes, was audio recorded, and transcribed verbatim. They provided insights into participants’ evolving experiences, collaborative learning, and responses to the sessions as they unfolded.

#### Semi-structured Interviews

Interviews allowed for a deeper understanding of how the sessions were experienced by care home staff and playwork practitioners, enabling reflection on both challenges and successes. They were guided by a semi-structured topic guide tailored to each participant group (playwork students, lecturers, and care home staff). Questions explored participants’ experiences of the sessions, perceptions of impact on residents, and suggestions for future improvements, as well as reflections on the relevance of playwork in dementia care settings.

Two in-person interviews (approximately 15 minutes each) were conducted with care home staff directly after they had attended a playwork session, to capture immediate reflections. Individual interviews were conducted via MS Teams approximately one month after the final session with the wellbeing coordinator, playwork students and lecturers. They lasted on average 60 minutes and were transcribed verbatim. The home manager could not be interviewed, as they had left their position prior to the study’s conclusion.

These longer interviews explored in more depth how the sessions influenced participants’ practices, perceptions, and professional development, particularly regarding their evolving understanding of dementia and the potential role of playwork within care settings.

#### Observations

The researcher attended four playwork sessions and collected written observations at a general, anonymous level. Due to ethical constraints, no data about residents was collected, and no identifying information about any individuals was recorded. Observations focused on the actions, interactions, and behaviours of the playwork students and care home staff. Notes included contextual information such as the types of activities taking place (e.g., games played, session structure), the number of participants present, and general dynamics in the room (e.g., staff-student collaboration, facilitation styles, and environmental cues that shaped the sessions).

### Data Analysis

Reflexive thematic analysis was conducted following Braun and Clarke (2024). It ensured that patterns and themes were identified in a rigorous yet flexible manner, allowing participant perspectives to guide the interpretation of findings. Inductive coding was conducted by the researcher. The thematic analysis was structured around two key areas: (1) approaches used during the programme and (2) perceived impacts on participants. The findings were shared with the playworkers and the care home wellbeing coordinator, who confirmed the relevance and accuracy of the analysis.

### Ethics

This study received ethical approval from Leeds Beckett Research Ethics Committee (ref:124,360), following UK research governance requirements. Research involving the direct collection of data from adults who lack capacity in the UK requires approval from a specialist national panel and a justification must be provided for including adults who lack capacity. These approvals require extensive documentation and typically take around six months to obtain. Given that this was a small-scale, time-limited pilot study designed to assess the feasibility of playwork in a care home setting, securing such approvals was not feasible. The feasibility of the playwork programme could be evaluated without collecting data directly from residents who lacked capacity.

## Findings

### Part 1: How can Playwork Be Delivered in Care Home Settings with People with Dementia?

This section examines the key playwork approaches used during the programme and how playworkers adapted them in working with care home residents living with dementia. It explores why the playworkers and the care home wellbeing coordinator believed these approaches were effective, and the challenges faced in applying them.

### Fostering Play that is Responsive and Participant-Led

A key principle of playwork is to be responsive and participant-led. This approach was used in the care home, allowing residents to guide activities.

We were being very flexible and adaptable […]. Coming in with a bit of a plan and then almost immediately changing that due to what we learn and what we saw that the residents were more or less engaged with. (Student 1, reflective diary, May 2024, session 4)

Playworkers offered diverse, open-ended materials that encouraged exploration. These items could be adapted to fit different spaces within the home and tailored to various abilities, helping residents to remain involved in the sessions on their own terms.

Resident 9 looked disengaged. I thought that this might be because they had thrown their beanbag at the skittles and no longer had anything to play with. To test this theory, I retrieved a beanbag, asked Resident 9 whether they wanted it and placed it in their hand. This had the immediate effect of reanimating them and they spent several minutes clasping the beanbag and enthusiastically spectating on the game. Their posture lifted, they sat forward in their chair and were smiling and laughing. I ensured that each subsequent time they threw their beanbag at the skittles I retrieved it for them and withdrew from the play frame to allow them to continue their own self-directed play. (Lecturer 2, reflective diary, April 2024, session 1)

Being responsive to individual needs required paying attention to non-verbal cues and observing residents’ body language.

The playwork concept of play cues — or serve-and-return responses — when working with the residents was particularly apt. Many of the residents used little verbal communication (at least to begin with) and so being attuned to and responding to subtle indicators such as eye-contact, and changes in body language and facial expression was important in ensuring that they were included and their play needs were met. (Lecturer 1, reflective diary, May 2024, session 4)

Getting to know the residents through regular attendance in the sessions allowed the playworkers to better understand these play cues.

Having a lot of the same people come back made us more comfortable and able to be like ‘oh, I kind of know what you like and how you interact. So I'm gonna be able to be a bit more comfortable to suggest things and to work with you’. (Student 3, interview, July 2024)

Playworkers had to learn to slow down, a significant shift from their usual practice of quickly switching activities to keep children engaged.

I was playing the same game with them for ages and it was so beautiful. But I’m used to children and they like to switch it up so I found myself getting a bit bored, but obviously they weren’t. (Student 2, interview, July 24)

### Adapting to the Care Home Space

Playworkers view spaces as dynamic environments that can be continually transformed based on the participants’ needs and interests. Although experienced in a variety of settings, they had never worked in a care home before and had to adapt activities to fit the available spaces — often adjusting on the spot.

The adaptability of playwork meant that, although we had to change rooms, this was fine, and it worked well — we just changed what we were going to do, we’re used to scrapping ideas. (Student 3, reflective diary, April 2024, session 2)

When we first arrived, we had to swap rooms from the large, open space we thought we would be using to a smaller, darker room upstairs. This was tricky both because of space but also because we had confused the residents by coming into the original space and starting to set things up and then having to pack up and leave five minutes later. The room change actually turned into a positive because it created a smaller space for communal activities, but it was difficult at the start. (Student 4, interview, July 2024)

### Balancing Support and Agency

Facilitating agency in a safe and supportive environment is a fundamental aspect of playwork. However, playwork theory also acknowledges that play inherently involves an element of risk — essential for meaningful engagement, exploration, and self-expression (Brown, 2014; Hughes, 2001). While care home environments often prioritise safety to the extent that opportunities for spontaneous engagement are restricted, playwork challenges this by emphasising a balance between protection and freedom. Throughout the programme, playworkers had to develop a nuanced understanding of when to step back and allow individuals space to take risks, rather than intervening too readily in the name of safety.

Once I’d realised to step back a bit, I observed Resident 3 and Resident 4 both reaching for beanbags that I would otherwise have picked up and returned to them. In allowing them to retrieve these things for themselves, it was evident from their facial expressions and nod of recognition that they gave to one another, that they felt they’d accomplished something significant. (Lecturer 2, reflective diary, May 2024, session 3)

Agency was supported through gross motor skills activities, which residents preferred over calmer, fine motor tasks.

Everything physical […] these kinds of activities just went down so well and they were just wanting to keep going and keep going with those games. (Lecturer 1, interview, July 2024)

Due to their limited experience with older adults, playworkers sometimes failed to recognise when residents were overexerting themselves and staff had to intervene.

The only thing that became a bit of a worry was when we were doing the game with the balloons and it was one of the care staff that said ‘Oh, we need to take it a bit easier with them.’ A resident was getting out of breath. […] And you suddenly thought, ‘Oh God, that could have gone very badly’. (Student 1, interview, July 2024)

### Creating a Platform for Free Expression and Exploration

Playworkers aim to provide children with the freedom to explore their ideas and emotions in a space that is non-restrictive. In the care home setting, this approach encouraged residents to fully express their personalities, allowing loud, competitive, and even destructive behaviour to coexist with shared adult humour.

I was standing with Resident 1 and Resident 2. Resident 1 accidentally threw a ball that landed on Resident 2’s chest. Resident 1 laughed and said, ‘You could have caught that’, to which she replied, ‘I did — just here’, pointing to her chest. Then Resident 1 joked that she should have opened her top to catch it down there instead. Resident 2 smirked for the first time — up until now, she had shown no facial expressions. (Lecturer 1, reflective diary, May 2024, session 3)

Resident 3 seemed to enjoy being a bit destructive. We got more of a reaction when she knocked down the cardboard boxes and they collapsed. (Student 2, diary entry, May 2024, session 4)

The wellbeing coordinator believed that the high energy and freedom in the sessions was one the key reasons residents engaged more with the playwork sessions than with the other activities offered in the home.

The music therapist came round and gave residents maracas and little balls that shake, and they weren't that bothered. But here, as soon as the playworkers arrive, their faces light up, I think it's definitely the energy […]. In this room, we can do it, we can throw things and smash things. I can throw that balloon and it's like this whole freedom thing, which I think everybody likes. (Interview, July 2024)

### Building Collaborative Relationships with the Care Home Staff

Collaborating with people and systems is crucial to supporting play. All playworkers stressed the importance of working collaboratively with care home staff before and during the project to ensure relevance, safety, and continuity once the programme ends. Yet, involving staff proved to be the most significant challenge. Although it was agreed that two staff members would support the weekly sessions, the care home was not able to provide this level of support.

There was no care staff, that was the biggest issue. (Wellbeing coordinator, interview, July 2024)

During the first two sessions, playworkers had to interact with the residents alone, as staff members who began to engage were called away.

From a staff point of view, because there was no consistency, it was really hard to know when they would come. (Lecturer 1, interview, July 2024)

Following the incident where a resident became breathless (*see 4.3*), the playworkers decided that future sessions would only proceed with a staff member present. From that day onward, the wellbeing coordinator became involved in all the sessions.

Having someone from the care home there was really useful because we could ask them about the residents and it just felt safer. (Student 3, interview, July 2024)

After witnessing the positive effects of playwork on residents, the wellbeing coordinator became an advocate for the project within the home. However, it was challenging to translate their experiences into terms that did not sound childish.

There were a couple of weeks where I found myself going out for a cigarette after the sessions. And the manager asked what they have done, then I went, ‘oh, it was really good…Well, we threw balls, we knocked boxes off’. And they're just looking at me. Daft. So it didn't really come across well. (Interview, July 2024)

One of the key challenges for the playworkers was communicating what the sessions would actually involve in practice. The term “playwork” initially raised concerns among care staff, who associated play with childhood and were trained (often through frameworks like Kitwood’s malignant social psychology) to avoid any practice that could be seen as infantilising. Without a clear explanation of how playwork supports autonomy, agency, and relational connection, the approach was misunderstood as potentially inappropriate. These concerns highlight the need to reframe play as a meaningful, age-inclusive form of engagement, rather than something inherently childish.

It just didn't sound dignified for people with dementia. Once we got into it, I totally understood the concept. But yeah…at first, using techniques you’d use with children on adults made me a bit sceptical about it. I thought ‘well, is that going to be dignified? and is that going to be age-appropriate?’. (Wellbeing coordinator, interview, July 2024)

Due to the lack of staff engagement, the project lacked continuity.

It’s important to have staff here to keep it going. It sort of proved it that it’s not happening now […]. But yeah, just to keep the legacy going so they could see how easy it was to engage with the residents and at what level and what the residents got out of it. And I think obviously, the staff missed out on that because they didn't see it. (Wellbeing coordinator, interview, July 2024)

A care staff member highlighted the importance of receiving training in playwork so to incorporate it into daily life at the home.

I’d love to be trained. So that we will get to do it for them on a daily basis, not just maybe once a while. It can be like an activity they can do after their breakfast or after their tea or something. (Interview, July 2024)

The wellbeing coordinator believed that, in future, management and staff would be more receptive if playworkers could provide a concrete demonstration of the programme before it begins.

I think, you know, telling them the case studies or showing them photographs or videos of how amazing it could be, and then saying, ‘You know you can achieve this — it’s really simple’, and then go into how they could do it […] maybe like with a staff training. You know, show them how good it can be. (Interview, July 2024)

### Part 2: What is the Perceived Impact of the Project?

The sessions led to a range of perceived impacts on residents, including improved emotional wellbeing, a stronger sense of recognition, increased social interaction, and greater engagement. They also prompted staff to reassess residents’ abilities and shifted students’ perceptions of dementia and care home life.

### Residents’ Emotional Wellbeing

The wellbeing coordinator observed that participating in the sessions improved residents’ moods.

We took everyone down as a group into the dining room. And they were a lot more cheerful. They were all laughing more than usual, and they were also all eating better. You know, their mood was better because of this and that translated to other people as well. Coming down from these brilliant sessions sort of took them through to the afternoon. (Interview, July 2024)

A care staff member noted a noticeable change in a resident who had never previously shown positive emotion at the care home.

Seeing her laughing and playing today… I was so surprised because I've never seen her like that before. (Interview, July 2024)

### Increased Sense of Recognition

Residents appeared to begin recognising the playworkers and anticipating the sessions as something enjoyable.

As soon as he saw you his face lit up and he saw the room, he knew what you were coming in for. (Wellbeing coordinator, interview, July 2024)

I remember as they walked in on the third week, they all seemed immediately kind of excited to see us, and I don't know if it was they remembered what was happening or just the memory of it feeling good but […] it didn't take as long to get people involved. (Student 2, interview, July 2024)

One resident unexpectedly remembered a playworker’s name when they arrived at the care home.

We had a resident who was downstairs, and she saw when the van first pulled up and [Lecturer 1] got out as well as everyone else, and she was looking out, having a nose there. And she went, ‘oh [Lecturer 1]'s there?’ And I went, ‘do you know [Lecturer 1]?’ And she said, ‘oh I've known them years.’ I'm like, ‘what?’. And it really confused me, but she recognised them and the name must have stuck. And that person usually doesn't remember. (Wellbeing coordinator, interview, July 2024)

### Social Interaction and Engagement

Playwork sessions appeared to foster positive, playful interactions among residents, enhancing group camaraderie and banter.

Resident 4 is so competitive and rallying the other residents up and there was a lot of like banter between them. (Student 1, group reflection, May 2024, session 2)

Resident 5 would sometimes throw the bean bag really hard and Resident 4 would raise his hand and pretend to protect himself... then when the bag hit him he would pretend and act out being mortally wounded, which Resident 5 would laugh at. (Lecturer 1, reflective diary, May 2024, session 3)

One resident who had previously been non-verbal and avoided interactions with staff and other residents began to engage during the session.

I've never even heard them speak, until last week when they spoke in the session […]. Their daughter nearly cried, and I nearly cried […]. Now they're holding themself up higher. They're smiling at people. They're trying to make conversation with them, get them joining. I'm convinced this is part of this project because this is where they come alive. (Wellbeing coordinator, interview, July 2024)

During the project, the playworkers diffused two small conflicts between residents.

Resident 3's racket came a little bit close to Resident 7's hand. Resident 7 was getting a bit distressed and angry about Resident 3 nearly hitting them. I reacted by acknowledging that what Resident 7 was seeing was happening. Resident 3 did not seem aware at all that Resident 7 was bothered or upset by this and they didn’t want to sit anywhere else. And so, I tried to make sure I was hitting it at either side of them so that I wasn't hitting it in the middle, so that the thing which was upsetting Resident 7, wouldn’t happen. So, I just tried to change so the situation didn't arise again […]. Resident 7 came down from being angry very quickly. (Student 2, reflective diary, May 2024, session 4)

The playwork sessions also appeared to have led to positive interactions between residents and some staff members, with some choosing to miss their break to continue playing.

We also had some members of the care home’s team come into the session […]. It seemed to take them a while to get into it but with some encouragement they became part of the play frame and engaged with the residents in a way I don’t think they had previously. Some even missed their break because they were having so much fun! (Student 1, group reflection, May 2024, session 4)

It helps them get to know you — not just as a carer. But for them to look at you and say, okay, this person is also there to listen, to play, to talk to. So this project, it’s very good. (Care staff, interview, July 2024)

The wellbeing coordinator also noted that the sessions started to influence broader activities within the home. For example, residents who took part in playwork started to increasingly participate in home-run exercise, while previously not interested.

We did an exercise session, and it was all the same people who had attended the playwork sessions. They came and it wasn’t like before, where they wouldn't really want to do it. ‘Not doing that’. but straight away this time they were smiling and laughing and making jokes out of it. I thought ‘you've never done that before’. (Interview, July 2024)

### Staff Re-assessment of Residents’ Abilities

Before the project began, the home manager warned the playworkers that most residents would likely be unable to stand or engage in gross motor activities due to physical limitations. However, it quickly became evident that the residents were more mobile and physically capable than the staff anticipated.

I had a brief conversation with a care staff member who explained that they hadn’t before seen Resident 4 get up from his chair unaided so easily. They were doing so with alacrity to retrieve and throw various balls at the skittles whilst laughing and smiling. (Lecturer 2, reflective diary, April 2024, session 1)

As the project progressed, residents displayed increased mobility and physical independence.

The physical capability and capacity of the residents to play actively using their bodies extended as they played more. Underarm throwing became overarm throwing; several residents became more mobile and moved from sitting to standing, and then from standing to moving to different parts of the room and sitting in different chairs. Games that initially involved rolling, throwing, or batting of balls, beanbags, shuttlecocks and balloons developed into games of kicking and heading. (Lecturer 2, reflective diary, May 2024, session 5)

It felt like there was this growing agency of the residents. The first week when we're doing the skittles, we were like, ‘oh the balls are out, we'll go get the balls’. And this time, I've definitely noticed they were doing it. They’re going and picking up the aeroplane that's been dropped and people are like reaching and picking up the balloons to get back involved. (Student 1, interview, July 2024)

These shifts suggest that earlier safety concerns, while well-intentioned, may have stemmed in part from underestimations of the residents’ physical capabilities. The playwork sessions offered an alternative space where movement, spontaneity, and playful risk-taking were encouraged. This allowed staff to observe residents not as fragile or passive, but as physically engaged, motivated, and capable of adapting to challenge. As a result of participating in the playwork programme, care staff noted that they had uncovered new aspects of the residents and began reassessing their capabilities. For example, one resident’s care plan was updated to acknowledge previously unrecognised physical abilities.

Because they were still a fairly new resident, we weren’t quite sure what they could do, what they couldn't do. You know, we have to sort of learn when we don't have much background information about them so I translated how active they were, standing up and throwing and sitting down and moving about. So they’ve put that in his care plan that he was much more mobile than they thought. (Wellbeing coordinator, interview, July 2024)

### Impacts on Playwork Students

All students’ perceptions of dementia and care homes shifted positively as a result of the project.

I had this kind of idea that care homes are a depressing place. I thought it would be quite depressing and people would be grumpy and maybe they'll be rude. But the care home we were at, all the staff were lovely, the residents were lovely, and it was surprisingly easy to get into it and to feel comfortable in the environment that we were in. (Student 1, interview, July 2024)

Before going in, I expected the residents to be more confused or angry […]. My perception has completely changed throughout this process, and my expectations are constantly being shattered. (Student 2, interview, July 2024)

Two students began exploring work with individuals living with dementia in care home settings as a potential career path.

It definitely made me think, ‘Oh, this is something I would want to do and get involved with more’. For sure, I would like to look at even just like volunteering, or I think at some point I would love even like a job. (Student 2, interview, July 2024)

I think it has definitely opened the door for me because going into it, I never worked really with anyone that wasn't a child, like all the jobs I had when I was young, it was working with kids. (Student 1, interview, July 2024)

## Discussion

This study is among the first to explore the feasibility of applying playwork approaches in a dementia care home and to assess their perceived impact on residents and playwork students. It makes several distinct contributions to knowledge.

This study places *play* itself at the heart of its inquiry rather than as a by-product or vehicle for other goals. In much of the existing dementia literature, play is rarely defined or examined as a concept in its own right; instead, it is often treated as an incidental or secondary outcome of arts-based or psychosocial interventions rather than the primary focus (George & Houser, 2014; Kontos et al., 2017). In contrast, this study approaches play as a distinct and purposeful mode of engagement, foregrounding its spontaneity, openness, and potential for co-constructed meaning. This approach aligns with Swinnen and de Medeiros (2018), who draw on Huizinga’s *homo ludens* to argue for play as a serious, meaning-making activity that resists instrumentalisation. They suggest that recognising the ludic capacities of people living with dementia challenges deficit-based narratives and affirms their agency, creativity, and relationality. By positioning play as both practice and principle, our research contributes a conceptual reorientation within the field of dementia care that treats play not as peripheral, but as central to human flourishing. Building on this conceptual reorientation, our findings demonstrate how playwork can be practically enacted with care home residents living with dementia in ways that respect their individuality, agency and freedom of expression.

Our findings demonstrate that playworkers can adapt their approaches, so they are appropriate for older adults living with dementia. Their responsive, participant-led methods are well-suited to this population because they allow for flexibility, accommodating various spaces, individual needs and abilities, even within a group setting. This resonates with findings from arts-based studies demonstrating that responsive activities can foster meaningful engagement by allowing residents with dementia to more inclusively take part and drive interactions (Basting and De Medeiros, 2014; Hatton, 2016; Kontos et al., 2017). This study therefore continues to address some of the concerns that play can be demeaning or infantilising for this population (Killick, 2013) by showing that, when well-facilitated, it positions the person living with dementia as a decision-maker and an active creator of meaning.

This study contributes to growing interest in relational models of agency that move beyond notions of autonomy-as-independence (Klein & Goering, 2023). In many care contexts agency is framed in terms of rational choice or independence, concepts that marginalise individuals with cognitive impairments or communication challenges (Boyle, 2014). Playwork offers a more expansive understanding of agency — as emergent, embodied, and co-constructed in the moment. These insights align with frameworks such as relationship-centred care (Nolan et al., 2004) and relational citizenship (Kontos et al., 2017), which argue that personhood is sustained and expressed through interaction rather than isolated cognition. Rather than aiming to preserve a coherent, pre-dementia identity, these models affirm the person as they are now. Playwork is well-positioned within this paradigm. It fosters environments that are permissive rather than prescriptive, validating spontaneous expressions and shared meaning-making between residents and facilitators. Agency, in this context, is not an individual possession but a relational accomplishment.

A further contribution of this study lies in its treatment of risk, not as a threat to be eliminated but as a necessary condition for meaningful engagement. Most dementia care interventions prioritise safety and structure, often seeking to reduce agitation (Hsiao et al., 2020) or improve cognitive function (Lizuka et al., 2018). While such aims are important, they can lead to overly risk-averse environments that limit spontaneity, exploration, and expression.

Playwork challenges this by recognising that play inherently involves risk. Playworkers in this study welcomed moments of chaos, experimentation, and humour. Residents particularly responded to activities that involved noise, movement, or competition — experiences that are often discouraged in traditional care, but proved vital to their engagement. As play theorists such as Hughes (2001) and Brown (2014) have argued, controlled risk becomes a pathway to affirmation, not danger**.** Our findings reinforce calls within dementia studies to reframe risk not as a liability but as an essential aspect of autonomy and flourishing (Killick, 2013; Marsh et al., 2018). In embracing some element of unpredictability, playwork can provide a model for care that supports experimentation, self-discovery, and freedom.

One challenge identified was the limited involvement of care staff, due to time pressures and understaffing. The wellbeing coordinator played a crucial role in sustaining the sessions, but most care staff were unable to attend regularly. This had implications for the long-term impact of the programme as without staff witnessing or participating in the play, there was little opportunity for the approach to embed within everyday care practice. However, when staff did engage, the impact was notable, with care staff reporting a shift in how they perceived and related to residents. These moments highlight the potential of playwork not just to support resident wellbeing, but to contribute to humanising the experience of care work. Play became a shared space where residents and staff could relate differently — less as carer and cared-for, and more as co-players in a relational moment. Staff engagement is therefore not necessarily essential for safe or meaningful play to occur, but it is vital for continuity. For playwork to have a lasting impact in dementia care settings, care teams must have opportunities to witness, reflect on, and experiment with these approaches.

Beyond its immediate value to existing staff, the programme also suggests opportunities to expand and diversify the care workforce by engaging a new generation of professionals. Interviews with playwork students revealed a significant shift in their perceptions of individuals living with dementia, and they expressed enthusiasm about working in care home settings in the future. This is consistent with other studies on creative intergenerational programmes in care homes (Danker et al., 2023; Lokon et al., 2017). In a climate where staff recruitment and retention remain challenging (Care Quality Commission, 2023), this programme could offer a promising new avenue for addressing workforce shortages.

Finally, this programme was conducted in a care home rated as requiring improvement and lacking some of the supportive structures that typically facilitate implementation — making the process more challenging, yet equally vital. Playwork, as a low-cost programme, has the potential to be both sustainable and feasible in such settings. The flexibility of this approach is particularly valuable, as it allows for adaptability in challenging situations, where more rigid programs might fail.

## Study Limitations

The programme was conducted at a single site, which may not reflect the conditions, culture, or practices of other care homes. The study was also conducted over a short timeframe, which may not have been sufficient to observe long-term outcomes. The small sample size limits the generalisability of the findings to other populations or settings. Only one care staff member was consistently involved throughout the project, with other staff participation being sporadic and difficult to capture.

Due to ethical clearance constraints, residents with dementia could not directly participate in the evaluation process. We acknowledge the limitations of this approach, particularly in capturing residents’ lived experiences and perspectives, as the research relied on the perceptions of playworkers and staff.

## Recommendations for Future Practice and Research

Future programmes should more closely consider what a relationship-centred approach to play looks like from a whole-home perspective, so that play becomes embedded in the everyday life of the home rather than tied to short-term projects. This resonates with the model of *relationship-centred care* that recognises the importance of positive relationships between people living with dementia, relatives and staff, but also considers the broader health and social care system, as well as the broader community in which the person lives (Nolan et al., 2004).

Building on calls from arts practitioners to bridge the gap between social care and creative practice (Basting & de Medeiros, 2014), it is essential to develop a shared language across disciplines and invest in tools and training rooted in lived experience. This approach is crucial for fostering meaningful, long-lasting impacts on residents, staff, and families. A whole-home approach would also allow for greater inclusivity, ensuring that residents from diverse backgrounds and with different physical or cognitive abilities can participate, not only those hand-picked for group activities. For instance, individuals who are bedbound or who prefer not to engage in structured sessions may still benefit from playful, relational encounters. Future research should explore how play, and the dynamics of facilitation and participation, may be influenced by cultural background, language, and identity.

Longer programmes, based on a residency model such as the University of Wisconsin-Milwaukee’s Student Artist in Residence programme (USA) or The Spitz Charitable Trust (UK) could provide an avenue to develop more organic and impactful programmes for all parties involved. It could also help develop more meaningful intergenerational connections and long-lasting positive attitudinal changes for playwork students, thereby strengthening the social care workforce.

Future research should also address the limitations of this study through a more extensive evaluation that includes ethical approval for collecting data directly from people living with dementia who lack the capacity to give informed consent. We acknowledge the broader ethical debate surrounding research involving people with dementia, particularly the critique of ethics guidelines that may inadvertently exclude certain voices due to overly rigid interpretations of capacity (Pieta & Diodati, 2023). While safeguarding protocols remain essential, it is equally important to reflect on ways to ethically and inclusively engage individuals with dementia in research, so their lived experiences can help shape care practice.

## Conclusion

Playworkers demonstrated the ability to adapt their methods to be appropriate and inclusive for older adults living with dementia in care homes. Playwork can promote agency and authentic self-expression, aligning with relational citizenship by enabling residents to actively participate in relationships through creative, embodied actions. The sessions were perceived to have positively impacted residents’ emotional wellbeing, sense of recognition, social interaction, engagement, and some staff members’ assessments of their abilities. Playwork may offer a promising avenue to strengthen the dementia care workforce by fostering meaningful connections between residents and care staff, while also attracting a new generation of workers. Collaboration with care homes is essential to maximising positive impact and ensuring long-term benefits for residents. Further research is needed to explore how playwork practices can be embedded sustainably within diverse care home ecosystems.

## Data Availability

Given the observational and reflective diary-based nature of the data, it is not feasible to sufficiently anonymise the content to ensure compliance with privacy standards. As a result, the data cannot be made publicly available.*
